# A 24-h restraint with food and water deprivation: a potential method to establish a model of depression in pigs

**DOI:** 10.3389/fvets.2023.1274497

**Published:** 2023-10-09

**Authors:** Sen Yang, Qiang Zheng, Guoan Yin

**Affiliations:** College of Animal Science and Veterinary Medicine, Heilongjiang Bayi Agricultural University, Daqing, China

**Keywords:** animal model, depression, pig, acute stress, 24-h restraint, food and water deprivation, animal welfare

## Abstract

Adverse stress, such as the long-term restriction of food intake and activity in intensive production, leads to a depression-like mental state in sows. Mood disorder, such as depression, is a widely concerned animal welfare issue. However, little is known about the biological mechanisms that underlie mood disorders in pigs. This study is the first attempt to establish a pig depression model by acute stress. A total of 16 adult Bama pigs were divided into the control and model groups, with 8 pigs (half male and half female) per group. The pigs in the model group were restrained for 24 h in a dark and ventilated environment, with food and water deprivation. After the restraint, behavioral tests (feed intake, sucrose preference test, open field test, and novel object test) were used to evaluate apparent indicators. The levels of COR and ACTH in the serum and the levels of 5-HT, NE, and BDNF in the hippocampus and medial prefrontal cortex were detected using ELISA to identify the physiological state. After acute stress, pigs exhibited decreased feed intake and sucrose preference, increased serum COR levels, decreased hippocampal 5-HT levels, and exhibited more fear. Finally, the model was evaluated according to the weight of the test indicators. The overall score of the model was 0.57, indicating that modeling was feasible. Although the reliability and stability require further verification, this novel model revealed typical depression-like changes in behavior and provided a potential method to establish a model of depression in pigs.

## Introduction

In intensive production, sows suffer from psychological and physiological stress as a result of long-term restriction of feeding and activity, herd transfer, diet change, barren environment and noise in pig farms, and other adverse conditions. Many sows have been found to be in a depression-like state. Stereotypic behaviors of sows are regarded as an external manifestation of depression-like symptoms with vacuum-chewing behavior being the most prominent (1). Studies have established that the frequency of stereotypic behaviors increased with the restraint time of sows (2). Simultaneously, depression-like changes also occur in the brain regions associated with emotion at the physiological and gene expression levels (3). These findings suggest that stress can trigger mood disorders in sows. However, the biological mechanism underlying depression in pigs remains unclear. However, there is no model of depression in pigs used to explore the pathogenesis and mechanisms of depression.

The ideal animal model of depression should meet three criteria: face, construct, and predictive validity (4). Face validity is a depression-like change in behavior and cognition (5). Studies have documented that body temperature, blood pressure, heart rate, and serum cortisol (COR) levels generally increase during or after acute restraint in stressed animals (6, 7), whereas aggressive conflict, motor activity, and exploration generally decrease (8). Construct validity means that model animals need to have the same pathophysiological characteristics as depressed individuals (9). Acute stress can induce dendritic remodeling and reduce phosphorylated actin in the medial prefrontal cortex (10). Additionally, the multiple effects of acute stress on the immune system and neuronal plasticity are demonstrated *via* changes in immunity, neurogenesis, cognition, and memory in animals exposed to restraint (11–13). Predictive validity mainly refers to the effect of antidepressant treatment on model animals. The establishment of rodent models of depression caused by acute stress can be deemed valid only by face and construct validity (14).

The study showed that 24-h restraint is successful in modeling depression in rats and could bring long-term effects. This gives us useful insight since long-term confinement and food and water deprivation are also stressful for pigs. Considering that pigs have strong stress resistance compared with rodents (15), we attempted to establish an animal model of depression in pigs by 24-h restraint with food and water deprivation. Moreover, the model was weighed for evaluation according to face and construct validity.

## Materials and methods

### Ethics statement

All experiments were approved by and conducted according to the guidelines of the Science and Technology Ethics Committee of Heilongjiang Bayi Agricultural University (DWKJXY2022039).

### Animals and feeding

Sixteen 6-month-old Bama pigs (8 males and 8 females) with uniform body weight (15 ± 1.15 kg) were randomly assigned to two groups (4 males and 4 females in the model or control group). Each pig was kept in a separate cage (80 × 60 × 60 cm).

Full-price pellet feed was fed once daily at 7:00 a.m., with free access to adequate food and water. Detailed reference: crude protein ≥ 15.0%, crude fiber ≤ 5.0%, crude ash ≤ 7.0%, calcium 0.5–1.5%, total phosphorus ≥ 0.5%, sodium chloride ≥ 0.3–0.8%, lysine ≥ 0.9%, and moisture ≤ 14.0%.

### Molding method

**Figure 1:**
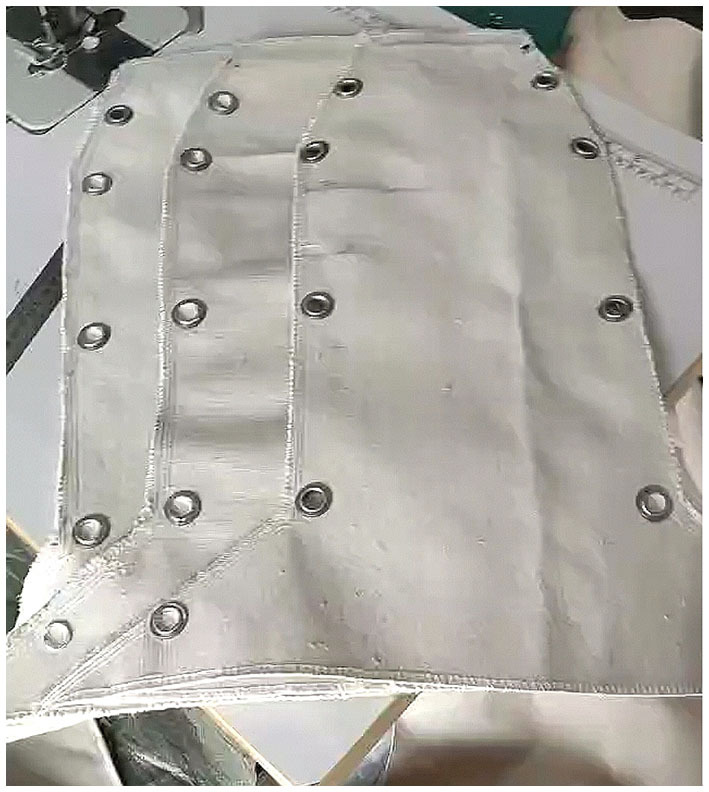
Restraint bag.

Each pig in the model group was restrained with canvas bags (Figure 1) to ensure that they could not move their limbs. From 7:00 a.m. on the first day to 7:00 a.m. on the second day, the pigs were restrained in a separate dark and ventilated room. During the restraint, the animals had no access to food and water. Once the restraint ended, pigs were immediately returned to their cages, with free access to food and water. Non-restraint pigs (the control group) remained in cages until the behavioral experiments started.

### Indicator collection

After modeling, the feed intake of each Bama pig was recorded, and the apparent indicators were evaluated by sucrose preference test (SPT) (16), open field test (OFT), and novel object test (NOT) (17). SPT provided two water tanks (10 L) containing water and 2% sucrose solution, respectively. The open-field arena measures 3 m × 3 m, and the enclosed metal wall is 1 m high. It is divided into 25 squares: 9 central areas and 16 peripheral areas. The behavior was recorded with a high-definition camera for 10 min, which was used to observe the number of squares pigs entered (squares entered), duration of time pigs spent in the periphery (time periphery), or central of the arena (time central), as well as the time of pigs idling (idle), nosing floor (nose floor), and nosing wall (nose wall) during OFT period. In NOT, the pig's reaction to an orange plastic rod (15 cm × 6 cm) was recorded within 10 min: latency period, frequency of contact with the object, and duration of contact with the object.

Then, blood samples were collected from the anterior vena cava of both groups. The blood was centrifuged at room temperature (2,000 rpm, 10 min) to obtain the serum. After all behavioral tests and blood collection, 2 male and 2 female pigs were randomly selected from each group for slaughter, and the hippocampus and prefrontal cortex were rapidly separated in a cryogenic chamber. Serum and brain samples were stored in a refrigerator at −80°C. Serum COR and adrenocorticotropic hormone (ACTH) levels, 5-hydroxytryptamine (5-HT), brain-derived neurotrophic factor (BDNF), and norepinephrine (NE) levels in the hippocampus and prefrontal cortex were detected using ELISA.

### Evaluation of statistical analysis and results

All data were analyzed using SPSS 16.0. Except for the sucrose preference data, all other behavioral test data were normally distributed, and the variance homogeneity test: *P* > 0.05. Except for the BDNF data of the hippocampus, all other physiological indicators were normally distributed, and the variance homogeneity test: *P* > 0.05. Independent sample *t*-test was used for indicators conforming to a normal distribution and non-parametric tests were used for indicators that did not.

The results are expressed as mean ± standard deviation. p<0.05 and p<0.01 were regarded as significant and extremely significant differences, respectively. Bar graphs were constructed using GraphPad Prism 6.0.

There is no significant gender effect was found among all indicators, so the gender effect is ignored.

Weight scoring rules (see Appendix for details) are as follows:

Combined with physiological indicators (Level I indicators), behavioral test indicators were regarded as apparent indicators (Level I indicators) for scoring the model. SPSS principal component analysis was performed on the Level II indicators of apparent and physiological indicators in the model, and principal components with eigenvalues >1 were selected.

R 4.2.1 was used to derive the contribution value of each Level II indicator to the selected principal components. The weight of each Level II indicator in the Level I indicator = (contribution value of principal component x1 /100) ^*^ (variance contribution rate of principal component x1 / total contribution rate) + (contribution value of principal component x2 /100) ^*^ (variance contribution rate of principal component x2 / total contribution rate) + (contribution value of principal component x3 /100) ^*^ (variance contribution rate of principal component x3 /total contribution rate). The weight score table of the apparent and physiological indicators of the model was calculated and obtained accordingly.

The score of indicator was set as 0, 0.5, and 1 for normal, significant, and extremely significant changes, respectively.

All the indicators of the animal depression model were quantified and integrated; the full score is 1. The score of each type of indicator after successful modeling was multiplied by the weight of the corresponding indicator and apparent and physiological indicators were added to calculate the total score when the depression model was successfully established. If the overall score was greater than 0.5, the model was considered successful.

## Results

### Apparent indicators

The feed intake and sucrose preference of Bama pigs in the model group were 526.87 ± 80.20 g and 35.97% ± 21.06, respectively, and in the control group, they were 723.36 ± 43.95 g and 88.66% ± 4.86, respectively. After modeling, both feed intake and sucrose preference were extremely significantly decreased in the model group (*P* < 0.01).

**Figure 2:**
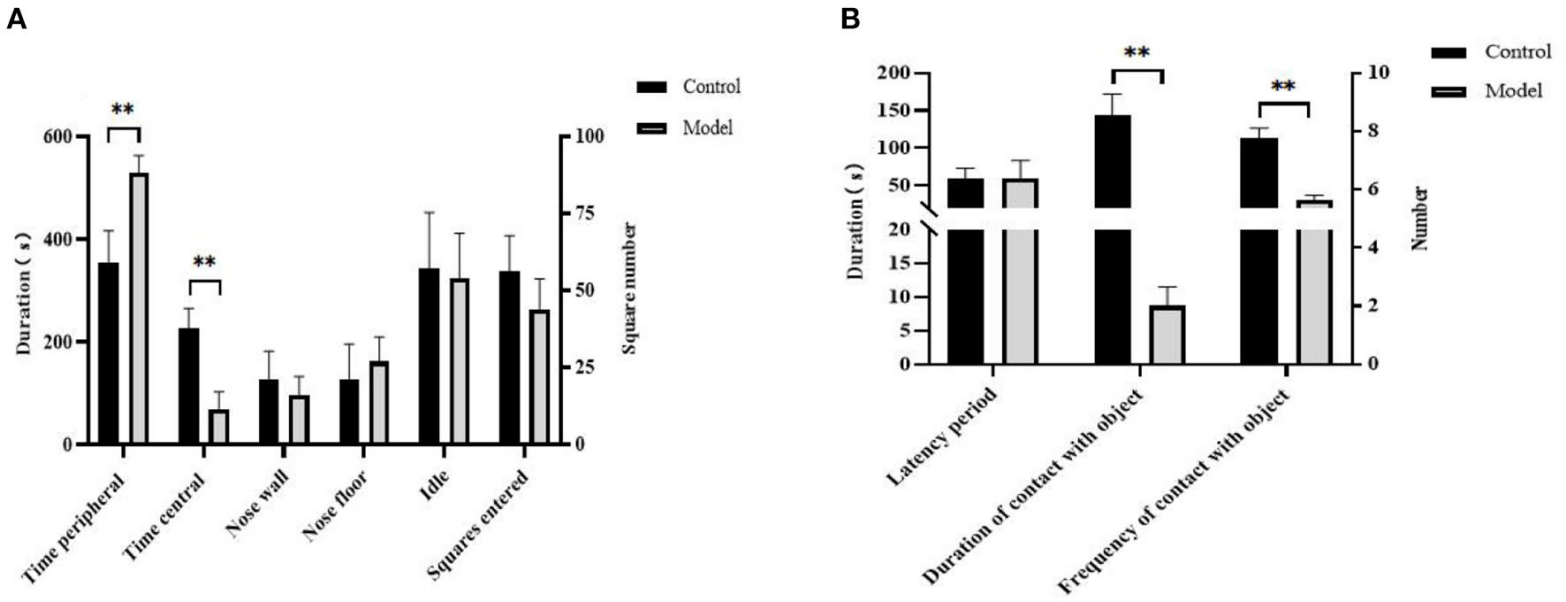
Changes in apparent indicators of Bama pigs after modeling. **(A)** Differences between the model and control group in open field test; **(B)** differences between the model and control group in novel object test. **P ≤ 0.01.

In the OFT, Bama pigs in the model group spent longer in the peripheral area (*P* < 0.01, Figure 2A), whereas in the NOT, they were exposed to toys less frequently (*P* < 0.01, Figure 2B) and for a shorter duration (*P* < 0.01, Figure 2B). There were no significant differences in the other behavioral indicators between the two groups (*P* > 0.05).

### Physiological indicators

After modeling, the serum COR levels in the model group were significantly higher than those in the control group (*P* < 0.05), and the hippocampus 5-HT level of the former was significantly lower than that in the latter (*P* < 0.05). There were no significant differences in other physiological indicators between the two groups (*P* > 0.05) (Table 1).

**Table 1:** Changes in physiological indicators of Bama pigs after modeling.

**Test indicator**		**Control**	**Model**	***P***
Serum	Cortisol (ng/mL)	274.22 ± 13.25	293.82 ± 9.74^*^	0.035
	ACTH (pg/mL)	147.17 ± 10.28	147.68 ± 8.44	0.447
Hippocampus	5-HT (ng/mL)	4.40 ± 0.16	4.04 ± 0.13^*^	0.022
	NE (ng/mL)	43.10 ± 1.10	42.20 ± 0.15	0.225
	BDNF (ug/mL)	0.67 ± 0.05	0.69 ± 0.03	0.230
Prefrontal cortex	5-HT (ng/mL)	4.58 ± 0.07	4.66 ± 0.06	0.179
	NE (ng/mL)	42.84 ± 1.99	41.52 ± 1.55	0.387
BDNF (ug/mL)	0.68 ± 0.02	0.69 ± 0.03	0.836

### Model evaluation

After the successful preparation of the acute stress animal depression model, the animals mainly show typical depression apparent phenomena such as anhedonia, restlessness, anxiety, panic, and decreased feed intake, whereas the changes in physiological indicators are small, and the model has certain limitations. Therefore, the apparent indicators were regarded as core indicators with a weight coefficient of 0.7, whereas the physiological indicators were regarded as directly related indicators with a weight coefficient of 0.3.

A total of two principal components for apparent indicators were selected, and the variance contribution rates were 48.418 and 30.273%, respectively. The cumulative variance contribution rate was 78.691%. The eigenvalues were 5.326 and 3.330, respectively (see Supplementary Table 1 in the Appendix).

A total of three principal components for physiological indicators were extracted, and the variance contribution rates were 37.588, 27.370, and 17.486%, respectively. The cumulative variance contribution rate was 82.444%. The eigenvalues were 3.007, 2.190, and 1.399, respectively (see Supplementary Table 2 in the Appendix).

The weight of the Level II indicators is shown in Table 2, and the overall score of the model is 0.57 (see Appendix for details).

**Table 2:** Weight of level II indicators.

**Level I indicators**	**Level II indicators**			**Weight**
Apparent indicator (weight coefficient:0.7)	Feed intake			0.100
	SPT			0.102
	OFT		Freq. squares entered	0.097
			Time peripheral	0.107
			Time central	0.107
			Nose wall	0.081
			Nose floor	0.085
			Idle	0.110
	NOT		Latency period	0.019
			Frequency of contact with object	0.094
			Duration of contact with object	0.098
Physiological indicator (weight coefficient:0.3)	Stress hormone		COR	0.131
			ACTH	0.132
	Neurotransmitter	5-HT	Hippocampus	0.136
			Prefrontal cortex	0.145
		NE	Hippocampus	0.121
			Prefrontal cortex	0.106
		BDNF	Hippocampus	0.079
			Prefrontal cortex	0.150

## Discussion

Some studies have documented that long-term restraint in intensive sow production develops various types of depressive symptoms such as stereotypic behavior, decreased sucrose preference, and pupil rigidity (18, 19). However, few have explored the pathogenesis and mechanisms of depression in pigs using animal models of depression induced by acute or chronic stress. We first established an acute depression model in minipigs using 24-h restraint along with food and water deprivation in a dark environment, and the results indicated that the effects on multiple apparent indicators were significant in Bama pigs.

Anhedonia is a core symptom of depression (20). After 24 h of acute restraint, the pigs in the model group exhibited extremely significant decreases in feed intake and sucrose preference, which have also been documented in humans and rodents. OFT is usually used to evaluate the autonomic behavior, exploration, and tension of animal models induced by stress, which reflects depression. The pigs in the model group spent more time in the peripheral area of the open field, but there was no significant variation in the activity, arch wall, and rooting. These behaviors were not accompanied by serious physiological abnormalities and recovered quickly. This indicates that only slight anxiety appears in pigs (16), which is also different from post-traumatic stress disorder. NOT is usually used to evaluate pigs' fear of novelty (21). In the NOT, the number and duration of exploring toys in the model group were significantly decreased, indicating that their inner fear increased. Pigs have stronger stress resistance. Research also determined that chronic social failure stress only leads to a short-term increase in salivary cortisol levels in sows, as a consequence of behavioral adaptations, and there is no sustained depression-like neuroendocrine effect (15). The increase in serum COR level and the decrease in 5-HT level in the hippocampus of those in the model group may be temporary acute responses to stress; however, there was no obvious depression-like neurological dysfunction. The study showed that 24-h restraint is successful in modeling depression in rats and could bring long-term effects (16), which also showed that pigs have a strong ability to resist stress.

The acute stress for this model mainly has an impact on the behaviors of Bama pigs, which is also recognized as the standard for successful modeling in rodents (22). To some extent, the behavior changes in Bama pigs proved that stress has brought about depression-like symptoms, and the modeling method is valid.

Gender is an important factor affecting depression modeling, and specific types of depression modeling methods may be more adaptable for either male pigs or female pigs. For example, the social defeat model of depression is more suitable for males (23), but the maternal separation model of depression is more suitable for females (24). Sows in farrowing crates for a long time exhibit depression-like behaviors, while there is little evidence of the psychological impact of activity restriction on boars. Both male and female pigs were used in this study, but no significant gender effect was found. However, it should be verified in future through large-scale experiments.

Buspirone, with partial 5-HT1A agonist properties, shows antidepressant-like effects, whereas ipsapirone, such as buspirone, a partial 5-HT1A agonist, is inactive (25). Because antidepressants are highly specific and the pathogenesis of pig depression is not yet clear, antidepressant drugs suitable for humans or rodents might not affect this pig model. Thus, an evaluation of predictive validity has not been conducted. Although the reliability of the model needs further verification, the overall score of 0.57 indicated that 24-h restraint with food and water deprivation is a potential method to establish a model of depression in pigs. As the first potentially feasible modeling method, it brings a new approach to research on the psychological disorder of pigs. Though optimizing the parameters and multidimensional model evaluation, this modeling method can provide a standardized and repeatable animal model for the study of the mechanism of depression in pigs.

## Data availability statement

The original contributions presented in the study are included in the article/Supplementary material, further inquiries can be directed to the corresponding author.

## Ethics statement

The animal study was approved by Science and Technology Ethics Committee of Heilongjiang Bayi Agricultural University. The study was conducted in accordance with the local legislation and institutional requirements.

## Author contributions

SY: Conceptualization, Formal analysis, Methodology, Validation, Writing—review and editing, Data curation, Investigation, Software, Visualization, Writing—original draft. QZ: Data curation, Investigation, Methodology, Writing—review and editing. GY: Methodology, Writing—review and editing, Conceptualization, Formal analysis, Funding acquisition, Project administration, Resources, Supervision, Validation.
