# Supplementary material for: A 24-h restraint with food and water deprivation: a potential method to establish a model of depression in pigs
Source: Front Vet Sci. 2023 Oct 9;10:1274497. doi: 10.3389/fvets.2023.1274497 (PMC10591077; doi:10.3389/fvets.2023.1274497)
Supplement: Supplementary file 1 [file Table_1.pdf]

Supplementary Material

1 Supplementary Tables

SPSS principal component analysis was performed on the Level II indicators of the apparent indicators in the model and control groups: feed intake, sucrose preference, open field test (total number of grids, peripheral zone, central zone, arch wall, rotting, idle), novel object test (incubation period, the number of exposure, the time of continuous exposure).

The variance decomposition table is shown in Table S1. A total of two principal components with eigenvalues ＞1 were selected. The variance contribution rates of two principal components were 48.418% and 30.273%, respectively, and the cumulative variance contribution rate was 78.691%. The eigenvalues were 5.326 and 3.330, respectively

Table S1 The variance decomposition table of the apparent indicator

Principal component	Initial eigenvalue			Extraction sums of squared loadings		
	eigenvalue	variance contribution rate(%)	cumulative variance contribution rate(%)	eigenvalue	variance contribution rate(%)	cumulative variance contribution rate(%)
1	5.326	48.418	48.418	5.326	48.418	48.418
2	3.330	30.273	78.691	3.330	30.273	78.691

The contribution value of each Level II indicator in apparent indicator to two selected principal components (with two decimal values) was obtained using the R 4.2.1 Facto Mine R as shown in Table S2. The weight of each Level II indicator in apparent indicator = (contribution value of principal component 1 /100) * (variance contribution rate of principal component 1 /78.691%) + (contribution value of principal component 2 /100) * (variance contribution rate of principal component 2 /78.691%). The final apparent indicator score is shown in Table S3.

Table S2 Apparent II indicator contribution value

	Principal component 1	Principal component 2
Feed intake	16.21	0.18
Sucrose preference	16.14	0.63
Total number of grids	0.24	24.80
Peripheral zone	17.19	0.35
Central zone	17.19	0.35
Arch wall	1.12	19.33
Rooting	1.50	19.63
Idle	0.09	28.40
Incubation period	0.06	4.76
The number of exposure	14.82	0.68
The time of continuous exposure	15.44	0.89
Aggregate	100	100

Table S3 Apparent indicator score

Grading (score)	Apparent indicator (weight coefficient: 0.7)										
			The open field test						The novel object test		
	Feed intake (0.100)	Sucrose preference (0.102)	Total grid number (0.097)	Peripheral zone (0.107)	Central zone (0.107)	Arch wall (0.081)	Rooting (0.085)	Idle (0.110)	Incubation period (0.019)	The number of exposure (0.094)	The time of continuous exposure (0.098)
1 (0)	Normal	Normal	Normal	Normal	Normal	Normal	Normal	Normal	Normal	Normal	Normal
2 (0.5)	Decrease ($P<0.05$)	Decrease ($P<0.05$)	Increase ($P<0.05$)	Increase ($P<0.05$)	Decrease ($P<0.05$)	Increase ($P<0.05$)	Increase ($P<0.05$)	Decrease ($P<0.05$)	Increase ($P<0.05$)	Decrease ($P<0.05$)	Decrease ($P<0.05$)
3 (1)	Decrease ($P<0.01$)	Decrease ($P<0.01$)	Increase ($P<0.01$)	Increase ($P<0.01$)	Decrease ($P<0.01$)	Increase ($P<0.01$)	Increase ($P<0.01$)	Decrease ($P<0.01$)	Increase ($P<0.01$)	Decrease ($P<0.01$)	Decrease ($P<0.01$)

SPSS principal component analysis was performed on the Level II indicators of physiological indicators in the model and control groups: serum COR, serum ACTH, hippocampal 5-HT, prefrontal 5-HT, hippocampal NE, prefrontal NE, hippocampal BDNF, and prefrontal BDNF. The variance decomposition table is shown in Table S4. A total of three principal components with eigenvalues ＞1 were selected. The variance contribution rates of the three principal components were 37.588%, 27.370% and 17.486%, respectively, and the cumulative variance contribution rate was 82.444%. The eigenvalues were 3.007, 2.190 and 1.399, respectively.

Table S4 The variance decomposition table of the physiological indicator

Principal component	Initial eigenvalue			Extraction sums of squared loadings		
	eigenvalue	variance contribution rate(%)	cumulative variance contribution rate(%)	eigenvalue	variance contribution rate(%)	cumulative variance contribution rate(%)
1	3.007	37.588	37.588	3.007	37.588	37.588
2	2.190	27.370	64.958	2.190	27.370	64.958
3	1.399	17.486	82.444	1.399	17.486	82.444

The contribution value of each Level II indicator in the physiological indicator to the three selected principal components was obtained using the R 4.2.1 Facto Mine R as shown in Table S5. The weight of each Level II indicator in physiological indicator = (contribution value of principal component 1 /100) * (variance contribution rate of principal component 1 /82.444%) + (contribution value of principal component 2 /100) * (variance contribution rate of principal component 2 /82.444%) + (contribution value of principal component 3 /100) * (variance contribution rate of principal component 3 /82.444%). The final physiological indicator score is shown in Table S6.

Table S5 Physiological II indicator contribution value

	Principal component 1	Principal component 2	Principal component 3
Serum COR	23.53	6.83	0.29
Serum ACTH	2.45	1.62	54.31
Hippocampal 5-HT	21.24	6.28	8.56
Prefrontal 5-HT	4.63	34.36	4.86
Hippocampal NE	18.45	4.02	11.18
Prefrontal NE	16.39	4.50	7.56
Hippocampal BDNF	8.78	10.60	1.92
Prefrontal BDNF	4.53	31.79	11.32
Aggregate	100	100	100

Table S6 Physiological indicator score

Grading (score)	Physiological indicator (weight coefficient: 0.3)							
	Stress hormone		Neurotransmitter					
			5-HT		NE		BDNF	
	COR (0.131)	ACTH (0.132)	Hippocampus (0.136)	Prefrontal cortex (0.145)	Hippocampus (0.121)	Prefrontal cortex (0.106)	Hippocampus (0.079)	Prefrontal cortex (0.150)
1(0)	Normal	Normal	Normal	Normal	Normal	Normal	Normal	Normal
2(0.5)	Increase ($P<0.05$)	Increase ($P<0.05$)	Decrease ($P<0.05$)	Decrease ($P<0.05$)	Decrease ($P<0.05$)	Decrease ($P<0.05$)	Decrease ($P<0.05$)	Decrease ($P<0.05$)
3(1)	Increase ($P<0.01$)	Increase ($P<0.01$)	Decrease ($P<0.01$)	Decrease ($P<0.01$)	Decrease ($P<0.01$)	Decrease ($P<0.01$)	Decrease ($P<0.01$)	Decrease ($P<0.01$)

All the indicators of the animal depression model were quantified and integrated; the full score is 1. The score of each type of indicator after successful modeling was multiplied by the weight of the corresponding indicator, and apparent and physiological indicators were added to calculate the total score when the depression model was successfully established. If the overall score was greater than 0.5, the model was considered successful. The overall score of the model is 0.57.
